# Victimisation in a French population of children and youths with autism spectrum disorder: a case control study

**DOI:** 10.1186/s13034-018-0256-x

**Published:** 2018-12-03

**Authors:** A. Paul, C. Gallot, C. Lelouche, M. P. Bouvard, A. Amestoy

**Affiliations:** 1 INCIA, CNRS, UMR 5287, Centre Ressource Autisme Aquitaine, Centre Hospitalier Charles Perrens, Bordeaux, France; 2 Present Address: Centre Hospitalier Côte Basque, Bayonne, France

**Keywords:** Victimisation, Autism spectrum disorder, Bullying, Juvenile Victimization Questionnaire, Anxiety, Post-traumatic stress disorder

## Abstract

**Background:**

Children and youths with autism spectrum disorder (ASD) have behavioural characteristics and severe social disabilities that make them vulnerable to victimisation. The current study explores the prevalence of peer victimisation in this population in France.

**Methods:**

We used the Juvenile Victimization Questionnaire—Screener Sum Version in a French sample of 39 children and youths with ASD and 53 typically developing (TD) children and youths and tested the association of the victimisation with socio-demographic factors and clinical factors of anxiety and post-traumatic stress.

**Results:**

The results indicate that 72% of the subjects with ASD had been victimised during the previous year and 94.9% during their entire lifetime. Of all students victimised at least once over the course of their lives, 75% had been victimised at school. Their peer victimisation score was significantly higher than in the TD group and was correlated to clinical factors such as a deficit in social skills and the severity of post-traumatic symptoms. Symptoms of anxiety were reported by parents of children and youths with ASD in 80% of cases.

**Conclusions:**

Children and youths with ASD are particularly vulnerable to victimisation at school. Discussion focuses on the importance of considering the impacts and needs of school integration of this population in France in order to prevent these phenomena and their consequences.

## Introduction

Autism spectrum disorder (ASD) is a public health priority [1]. This term refers to a set of heterogeneous neurodevelopmental conditions, characterised by early-onset difficulties in social communication, along with unusually restricted, repetitive behaviour and interests. The term “spectrum” refers to the wide range of symptoms, skills, and levels of impairment that people with ASD can have. ASDs are characterised by communication deficits, such as responding inappropriately in conversations, misreading nonverbal interactions, or having difficulty building friendships appropriate to their age. In addition, people with ASD may be overly dependent on routines, highly sensitive to changes in their environment, or intensely focused on inappropriate items or on unusual patterns of interests. Again, the symptoms of people with ASD will fall on a continuum, with some individuals showing mild symptoms and others having much more severe ones [2].

The worldwide population prevalence of ASD from recent studies is about 66/10,000 (0.66% or 1 child in about 152 children with a diagnosis of ASD) [3, 4], with an approximate male-to-female ratio of 5:1. Comorbidity is common in this population (more than 70% have concurrent conditions) [5]. However, there is a lot of variability in the reported prevalence of ASD in children and youths. Some recent studies have shown prevalence rates that are 2 to 4 times higher, for example Kim et al. [6], who reported an ASD prevalence of 2.64%.

Theory of mind is the ability to attribute mental states (beliefs, intents, desires, emotions, knowledge) to oneself and to others, and to understand that others have beliefs, intentions, and perspectives that are different from one’s own. Theory of mind is crucial for everyday human social interactions and is used when analysing, judging, and inferring others’ behaviours [7].

Executive function comprises a set of cognitive control processes such as planning, cognitive flexibility, shifting attention, sustained or selective attention and response inhibition which regulates lower levels of cognitive processes (e.g. perception, and motor responses), thereby enabling self-regulation and self-directed behaviour toward a goal. This allows a person to break out of habitual behaviour patterns, make decisions and evaluate risks, plan for the future, prioritise and sequence actions, and cope with novel situations. ASD subjects show difficulties in these domains, which leads to difficulties in social adaptation (e.g. lack of initiative, ignorance of social codes, misunderstanding of intention in communication) [8].

Atypical processing is also reported in people with ASD [9, 10]. Various results demonstrated superior performances on several visuospatial tasks where local or detailed information processing is advantageous [11, 12]. Results indicate that an atypical early bias for detailed spatial information (“enhanced perceptual theory” of Mottron et al. [9, 10]) in ASD may affect development of facial and emotional recognition primarily involved in global processing [13].

Such atypical cognitive profiles (impaired social cognition i.e. deficit in theory of mind and social perception, executive dysfunction and atypical perceptual and information processing) may occur to varying degrees in individuals with ASD. These characteristics could make individuals with ASD more vulnerable to being victimised. On the one hand, vulnerability to peer victimisation, bullying and ostracism may be increased by socio-communicative and behavioural difficulties with peer interactions [14–16]. On the other hand, vulnerability to physical and sexual abuse may be related to intelligence quotient and to the difficulties in detecting the intentions of others [15]. Individuals with ASD are more likely than TD individuals to be socially withdrawn, which often leads to isolation and loneliness that continues into adulthood [17]. Such isolation then increases the risk of peer victimisation, as many of these individuals do not have the protective factor of supportive peers [14].

The features of ASD coincide with victimisation risk factors described in the victimology literature: young age, male sex, social disability, social stigma, carelessness, lack of vigilance, immoderate trust in the honesty of others, failure to report on endured offences, and social isolation [18, 19]. In this literature, peer victimisation, especially bullying, arouses keen interest [20, 21]. Bullying is a form of victimization characterised by repeated attacks of one or more children or youths on another for a variable duration; it can be physical, verbal or relational (exclusion) [22].

Children and youths with autism may also be targeted for abuse by sexual offenders. Mandell et al. [23] collected data from 1997 to 2000 on 156 children with autism. Caregivers reports indicate that 18.5% of children with autism had been physically abused and 16.6% had been sexually abused during their life. The rates of sexual abuse for children with developmental disabilities are almost two times greater than for typically developing (TD) children and the effects of sexual abuse may be exacerbated by social isolation and alienation [24].

Another type of victimisation that children and youths with autism can suffer is maltreatment. In a recent study on an adult population, ASD participants were 4 times more likely to report having experienced a form of maltreatment as children (including physical abuse, and psychological or emotional abuse from adults), compared to the control group. In the same study, ASD participants were also 27.1 times more likely to report having been teased by peers, 3.7 times more likely to report having been bullied by peers, and 7.3 times more likely to report having experienced sexual assault by a peer compared to control participants [25]. In another previous study, maltreatment was self-reported by 88% of a population of 180 parents of children with autism. This study showed that the risk of severe maltreatment increases with age and the severity of ASD [26].

Children and youths with ASD have difficulties building interpersonal relationships [2, 27, 28], which is a risk factor for victimisation [29] and can significantly affect their quality of life [14]. They also may have salient comorbid psychological symptoms (e.g. clinically significant anxiety) and intense behavioural and emotional responses to their environment which may place them at an increased risk of being victimised [30–32]. Recent research has shown that peer victimisation is associated with internalising symptoms such as withdrawal, somatic complaints, and anxiety/depression [33].

In France, school is compulsory for children aged 6 to 16 years. The educational system is under the authority of the Ministry of National Education. This system is divided into several levels: primary level (years 3–10: kindergarten and elementary school), secondary level (years 11–18: middle school and high school), and professional level, apprenticeships and college, with variable durations. In schooling institutions, there are one or several classrooms for each level. Public school institutions are free of charge while fees are charged for private school institutions.

In France, the number of students with ASD attending school has risen sharply. In 2008–2009, there were more than 12,000 students with ASD enrolled in mainstream school. In 2015–2016, 29,326 students with ASD attended mainstream school, which was an increase of 2.5 times compared to 2008 [34]. Studies in other countries have found high rates of peer victimisation and exclusion (up to 92%) in this group of students [35], compared to the general population (36.5% physical bullying and 13.7% relational bullying) [36]. In France, 11% to 12% of all children experience peer victimisation in a general education setting, according to a study of the Ministry of National Education [37].

In a recent meta-analysis, Maïano et al. [38] estimated the prevalence of general school peer victimisation among children and youths with ASD to be around 44%. Zablotsky et al. [39] reported that up to 63% of children and youths diagnosed with ASD may have experienced peer victimisation once in their lives and that the risk is higher in less protected, general education settings with TD peer classmates.

Given the great number of victimisation risk factors in children and youths with ASD, looking for rates of victimisation and poly-victimisation is particularly relevant. Poly-victimisation refers to the experience of multiple types of victimisation, such as sexual abuse, physical abuse, peer victimisation and exposure to family violence, not just multiple episodes of the same type of victimisation [40]. As for bullying, it is a form of victimisation that has an impact on academic achievement, school commitment, and dropping out [35]. It is therefore essential to assess this in the French population in order to adapt school preventive policies and increase focus on this issue.

The main objective of this study was to determine the prevalence of victimisation and poly-victimisation in a French population of children and youths with ASD compared with a control group of TD children and youths. Secondary objectives were to assess the association between such victimisation and socio-demographic (age, gender) attributes, or deficits in social skills in a sample of children and youths with ASD.

## Materials and method

### Participants

Participants with ASD (the “ASD” group) were recruited from patients diagnosed at an ASD expert centre in Bordeaux, France.

Regarding the ASD group, inclusion criteria were: having received mainstream schooling for at least 1 year, aged between 7 and 18 years, ASD diagnosis validated by a threshold score on the Autism Diagnostic Interview-Revised (ADI-R) [41] and the Autism Diagnostic Observation Schedule-Generic (ADOS-G) [42] and parents’ written consent provided. Exclusion criteria were: intellectual disability (ID; IQ < 70 on the WISC-IV [43]) and known neurological or psychiatric comorbidities, except attention deficit hyperactivity disorder (ADHD), in order to minimize confounding. Psychiatric conditions are known to be risk factors for victimisation [44]. ADHD was not excluded as it is a well-known and frequent comorbidity in children and youths with ASD [5].

French school authorities allowed us to recruit control participants from nine randomly selected classrooms of one private regular school institution in Châteauroux (Indre, France), representing a total of 250 families. The headmaster and governing board’s ethical approval was obtained.

Regarding the control group, inclusion criteria were: aged between 7 and 18 years and parent’s written consent provided. We were not allowed to include students under the age of 7 years. Exclusion criteria were: diagnosis of ASD, ID, and other known developmental, neurological or psychiatric disorders except ADHD. The absence of exclusion criteria was verified by questioning the parents.

The two groups were frequency-matched for age and sex and all of the participants had a good level of vocal verbal ability.

### Measures

In both groups, all questionnaires were administered by a psychiatrist to the children and youth’s parents during face-to-face or telephone interviews lasting for 20 to 30 min. We were not allowed to perform the assessments with children and youths in this study by the French school authorities for ethical reasons.

We used the Juvenile Victimization Questionnaire—Screener Sum Version (JVQ). This is a structured questionnaire inventorying victimisation and major forms of aggression during childhood [45]. It explores a wide range of events including non-violent victimisation that children, youths and their parents do not typically see as offences or crimes, such as neglect or emotional bullying. The JVQ reports on 34 forms of offences against children and youths that cover five general areas of concern: conventional crime (robbery, personal theft, vandalism, attempted or threatened assault, physical assault, bias attack, and kidnapping), maltreatment, victimisation by peers and siblings, sexual victimisation and witnessing (exposure to violence). Sample questions of the JVQ are given in Table 1.

**Table 1:** Sample questions from Juvenile Victimisation Questionnaire—Screener Sum Version

Conventional crime	In the last year, did anyone use force to take something away from your child that your child was carrying or wearing?
Maltreatment	Not including spanking on your child’s bottom, in the last year, did a grown-up in your child’s life hit, beat, kick, or physically hurt your child in any way?
Victimisation by peers and siblings	Sometimes groups of kids or gangs attack people. In the last year, did a group of kids or a gang hit, jump, or attack your child?
Sexual victimisation	In the last year, did a grown-up your child knows touch your child’s private parts when they shouldn’t have or make your child touch their private parts? Or did a grown-up your child knows force your child to have sex?
Witnessing	In the last year, did your child SEE a parent get pushed, slapped, hit, punched, or beat up by another parent, or their boyfriend or girlfriend?

A Francophone validated version used in a Canadian study was chosen because there is no French validated version of the JVQ [46].

The JVQ can be scored in a variety of ways to produce variables that are of interest for a number of different contexts. The most basic scores are item-level scores and module scores. We scored the JVQ by counting the number of reported victimisations over a lifetime and within the past year. We also used module sub-scores in order to assess each subtype of victimisation. The maximum score for each subtype of victimisation is 8 for conventional crime, 4 for maltreatment, 6 for victimisation by peers and siblings, 7 for sexual victimisation and 9 for witnessing. We standardised the averages of the sub-scores in order to compare them.

Regarding the screener sum version of the JVQ, poly-victimisation refers to five or more victimisation types within the past year and 11 or more victimisation types over a lifetime. This is different from levels of victimisation that refer to the total JVQ score. Low poly-victimisation refers to 5-to-7 victimisation types within the past year and high poly-victimisation refers to eight or more victimisation types within the past year [47].

Parents were asked to specify the main location of all reported victimisation events: at home only, at school only, both at home and at school or elsewhere.

We created a questionnaire to assess the clinical and forensic consequences of the victimisations. It was administered to the child’s parents whenever there was a positive answer to at least one question on the JVQ. This questionnaire explored the presence of signs of stress such as symptoms of anxiety, depression, eating disorder, addictive behaviours, self-aggressive or suicidal behaviours (“Since the event(s) during which your child was victimized, have you or others who have cared for your child identified one or more of the following symptoms: your child replays the victimizing events in his/her games or activities; your child has attention or concentration problems affecting his/her schooling…”). According to Vila et al. [48], such signs may be the consequences of a psychological trauma such as victimisation in children and youths. The stress level of the participants was assessed by counting the number of parents who reported signs of stress. This questionnaire also assessed the number of complaints filed following a victimisation (“If the victimizing event or events involved one or more offenders, has the perpetrator or perpetrators ever been the subject of a complaint, fine or criminal prosecution?”).

Because there is no French validated scale assessing children and youths’ post-traumatic stress disorder (PTSD) symptoms in a rater-administered form, we chose to use the post-traumatic stress disorder CheckList-Scale (PCL-S) which is one of the most well-known and commonly used scales for assessing PTSD in France [49]. It is a self-administered questionnaire measuring three major sub-syndromes of PTSD (repetition syndrome, avoidance and autonomic hyper-arousal). The PCL-S was adapted by the authors into a caregiver version in order to be administered to the parents of participants. It was used due to its good empirical validity and its stability over time (test–retest reliability of 0.96) [50]. However, this version of the PCL-S was not validated.

Deficit in social interaction has a role in the occurrence of victimisation [51]. It was assessed in the ASD group using a French validated version of the social responsiveness scale (SRS). It is a parent and/or teacher rating scale of 65 items about a child’s ability to engage in emotionally appropriate reciprocal social interactions. Its internal consistency (0.91–0.97), test–retest reliability (0.84–0.97), inter-rater reliability (0.76 and 0.95) and convergent validity with the Autism Diagnostic Observation Schedule as well as the Autism Diagnostic Interview-Revised and Social Communication Questionnaire (0.35–0.58) are good [52, 53].

The presence of ADHD in the ASD group had been previously verified according to the Diagnostic and Statistical Manual (DSM)-5 criteria at the ASD expert centre in Bordeaux, France. The cognitive profile was defined with the WISC-IV scale [43].

Socio-demographic factors such as age may play a role in the occurrence of victimisation in children and youths with ASD [14, 54]. For all participants, socio-demographic data were collected in order to assess the association between such data and victimisation: age, gender, the parents’ marital status, the subjects’ type of schooling (regular or specialised) and the presence of an individual teaching aid.

### Statistical analysis

SPSS Statistics version 17.0 was used for all statistical analyses. We performed a univariate analysis to calculate valid data, mean and standard deviation (SD). The Pearson or Spearman correlation test was applied respectively for pairs of parametric or non-parametric quantitative variables. Student’s *T* test with Welch correction or Wilcoxon-Mann–Whitney’s test was applied respectively for pairs of parametric or non-parametric variables including both a qualitative and quantitative variable. The Chi square test was used for pairs of qualitative variables only. Significance threshold *p* was set at 0.05 for all statistical tests.

## Results

### Population

Ninety-two children and youths—78 boys and 14 girls—aged 7 to 18 years were included in the study. The characteristics of the population are presented in Table 2. In the group of 39 individuals with ASD, 84.6% were male with a 5.5:1 male-to-female ratio. The age of participants was between 8 and 18 years and the mean age was 13.23 years (SD = 2.96). Half (53.8%) met the criteria for an ADHD co-occurring condition. Fifty-five percent were in a specialised classroom and 71.8% had an individual teaching aid. No ASD students were in a specialised classroom with an individual teaching aid. In the group of 53 control individuals, 84.9% were males with a 5.6:1 male-to-female ratio. The age of the control group ranged from 7.6 to 18 years and the mean age was 12.82 (SD = 2.49). No parents declared the presence of ADHD in the control group.

**Table 2:** Demographics and clinical characteristics of the population

	ASD (n = 39)	Controls (n = 53)
Age (years)	13.23 (2.96)	12.82 (2.49)
Sex		
Male	33 (84.6%)	45 (84.9%)
Female	6 (15.4%)	8 (15.1%)
Marital status of parents		
Living as a couple	31 (79.5%)	43 (81.1%)
Separated	8 (20.5%)	10 (18.9%)
Type of school		
Mainstream with a school aid	28 (71.8%)	
Mainstream in a specialised classroom	22 (56.4%)	
ADHD comorbidity	21 (53.8%)	
Intelligence quotient		
Verbal comprehension index	92.74 (25.45)	
Fluid reasoning index	94.18 (14.66)	
SRS (T-score)	76.50 (10.71)	

The data are expressed as mean (SD) or absolute value (percentage)

*ADHD* attention deficit hyperactivity disorder, *ASD* autism spectrum disorder, *SRS* social responsiveness scale

### Victimisation (JVQ scores)

**Fig. 1:**
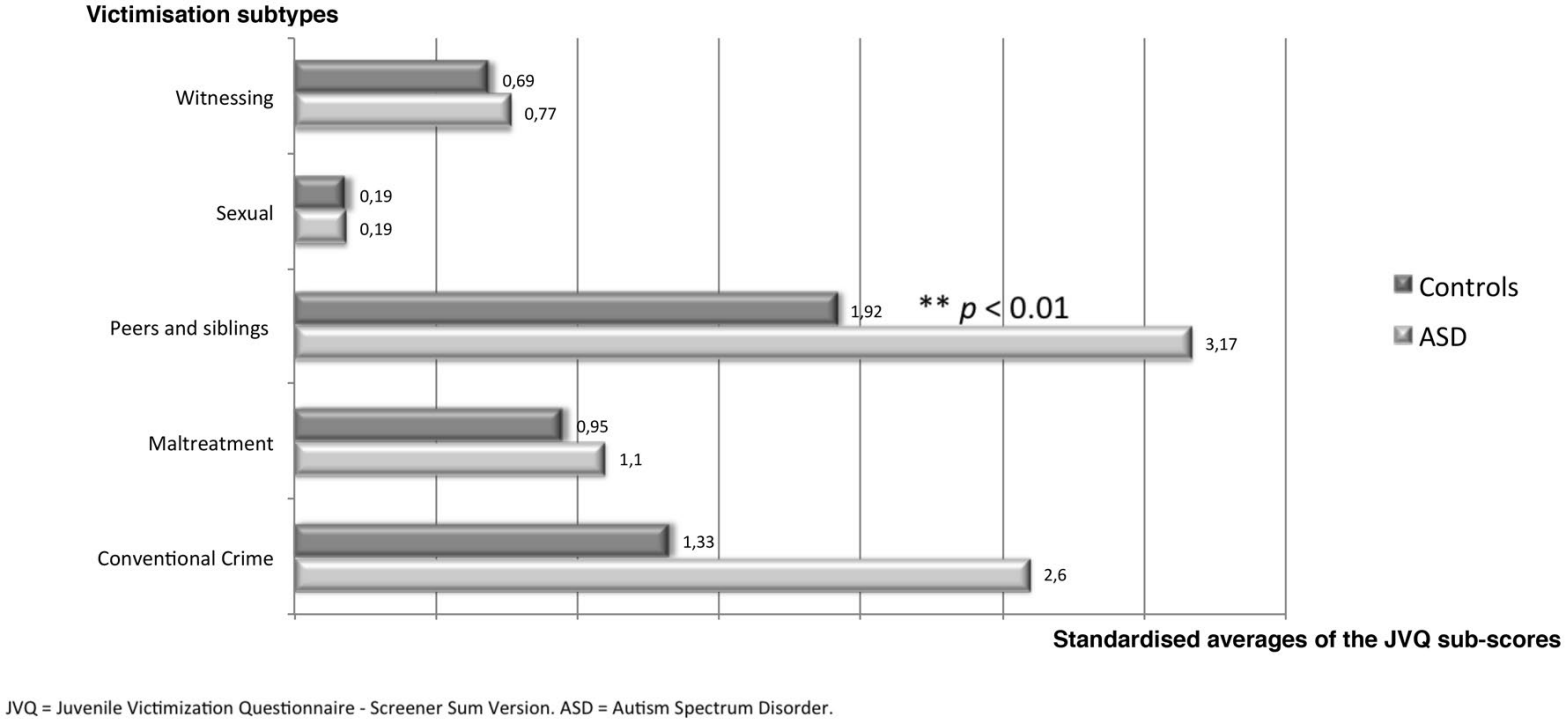
Lifetime victimisation sub-scores in the ASD and control groups

Among the participants with ASD, 71.8% (28 of 39) had experienced at least one victimisation event in the 12 months prior to this study, compared to 58.5% (31 of 53) in the control group. The difference was not significant. Over an entire lifetime, 94.9% (37 of 39) of ASD subjects had experienced at least one victimisation event, of any type, compared to 86.8% (46 of 53) in the control group, but no significant difference was found. On average, the total score of the JVQ over a lifetime was significantly higher in the ASD group compared to the control group (5.23 ± 3.42 versus 3.89 ± 3.23, *p* < 0.05). Among participants with ASD, 87.2% (34 of 39) had been victimised at least once by their peers or siblings during their life and 53.8% within the previous year (67.9% and 39.6% in the control group, respectively); the difference was not significant. On average, the JVQ sub-score assessing victimisation by peers and siblings was significantly higher in the ASD group compared to the control group (1.9 ± 1.23 versus 1.15 ± 1.03, *p* < 0.01) (Fig. 1).

In the ASD group, 23.1% (9 of 39) of the subjects were poly-victims compared to 17% (9 of 53) of the control group, but no significant difference was found. Assault with a weapon, bullying and emotional bullying were significantly more frequently reported in the ASD group than in the control group (Table 3). Twenty-eight ASD subjects had been victimised at school (75.7%), compared to only 6 (16.2%) at home and 3 (8.1%) elsewhere.

**Table 3:** Compared percentages of victimisation types (lifetime)

	ASD (n = 39)	Controls (n = 53)
Robbery	4 (10.3%)	3 (5.7%)
Personal theft	9 (23.1%)	18 (34.0%)
Vandalism	10 (25.6%)	13 (24.5%)
Assault with weapon**	9 (23.1%)	2 (3.8%)
Assault without weapon	22 (56.4%)	26 (49.1%)
Attempted assault	15 (38.5%)	15 (28.3%)
Kidnapping	0 (0.0%)	1 (1.9%)
Bias attack	12 (30.8%)	7 (13.2%)
Physical abuse by caregiver	3 (7.7%)	6 (11.3%)
Psychological/emotional abuse	13 (33.3%)	11 (20.8%)
Neglect	1 (2.6%)	1 (1.9%)
Custodial interference/family abduction	0 (0.0%)	2 (3.8%)
Gang or group assault	4 (10.3%)	4 (7.5%)
Peer or sibling assault	26 (66.7%)	32 (60.4%)
Nonsexual genital assault	2 (5.1%)	2 (3.8%)
Bullying***	18 (46.2%)	6 (11.3%)
Emotional bullying*	23 (59%)	17 (32.1%)
Dating violence	1 (2.6%)	0 (0.0%)
Sexual assault by known adult	1 (2.6%)	0 (0.0%)
Nonspecific sexual assault	0 (0.0%)	0 (0.0%)
Sexual assault by peer	0 (0.0%)	0 (0.0%)
Rape: attempted or completed	0 (0.0%)	1 (1.9%)
Flashing/sexual exposure	1 (2.6%)	3 (5.7%)
Verbal sexual harassment	3 (7.7%)	3 (5.7%)
Witness to domestic violence	1 (2.6%)	2 (3.8%)
Witness to parent assault of sibling	2 (5.1%)	3 (5.7%)
Witness to assault with weapon	5 (12.8%)	6 (11.3%)
Witness to assault without weapon	11 (28.2%)	14 (26.4%)
Burglary of family household	6 (15.4%)	7 (13.2%)
Murder of family member or friend	2 (5.1%)	0 (0.0%)
Witness to murder	0 (0.0%)	0 (0.0%)
Exposure to random shootings, terrorism, or riots	0 (0.0%)	1 (1.9%)
Exposure to war or ethnic conflict	0 (0.0%)	0 (0.0%)

The data are expressed as absolute value (percentage)

*ASD* autism spectrum disorder

* Significance threshold p < 0.05. ** Significance threshold p < 0.01. *** Significance threshold p < 0.001

### Clinical and forensic consequences of victimisation

Following the victimisation events, symptoms of anxiety, depression, eating disorders, addictive behaviours, and self-aggressive or suicidal behaviours were identified by 79.5% of parents of children and youths with ASD, compared to 69.8% for parents in the control group. The difference was not significant. On average, parents reported a significantly higher number of signs of stress for their children in the ASD group (4.5 ± 3.4 versus 2.3 ± 2.6, *p* < 0.01). Symptoms of PTSD including flashbacks, avoidance behaviours, insomnia, hypervigilance, attention or concentration problems and social isolation were found in significantly higher numbers in the ASD group than in the control group (Table 4). For subjects victimised at least once in their life, the mean PCL-S score of children and youths with ASD was significantly higher than that of non-ASD subjects (29.4 ± 9.7 versus 20.7 ± 4.3, *p* < 0.01). According to the score threshold set at 44 [55], three victimised ASD subjects (8.6%) had a diagnosis of PTSD, while there were none in the control group; the difference was not significant. No parents filed a complaint following victimisation.

**Table 4:** Compared percentages of repercussion symptoms of victimizations

	ASD (n = 39)	Controls (n = 53)
Flashback*	13 (33.3%)	6 (11.3%)
Putting back into action in games or activities	4 (10.3%)	2 (3.8%)
Nightmares	10 (25.6%)	8 (15.1%)
Emotional anaesthesia	5 (12.8%)	1 (1.9%)
Anhedonia	1 (2.6%)	2 (3.8%)
Pessimism	13 (33.3%)	10 (18.9%)
Avoidance behaviour*	12 (30.8%)	6 (11.3%)
Selective amnesia of facts	10 (25.6%)	12 (22.6%)
Irritability	13 (33.3%)	11 (20.8%)
Insomnia*	15 (38.5%)	9 (17.0%)
Hypervigilance*	9 (23.1%)	3 (5.7%)
Attention or concentration problems**	14 (35.9%)	5 (9.4%)
Somatic complaints	4 (10.3%)	6 (11.3%)
Enuresis	3 (7.7%)	0 (0.0%)
Resumption of thumb sucking	1 (2.6%)	2 (3.8%)
Sadness	5 (12.8%)	2 (3.8%)
Self-deprecation	14 (35.9%)	12 (22.6%)
Anxiety	11 (28.2%)	8 (15.1%)
Addictions	0 (0.0%)	2 (3.8%)
Risk behaviour	3 (7.7%)	5 (9.4%)
Social isolation*	7 (17.9%)	2 (3.8%)
Eating disorder	3 (7.7%)	4 (7.5%)
Scarification	2 (5.1%)	2 (3.8%)
Suicide attempt	3 (7.7%)	2 (3.8%)

The data are expressed as absolute value (percentage)

*ASD* autism spectrum disorder

* Significance threshold p < 0.05. ** Significance threshold p < 0.01

### Variables associated with victimisation (Table 5)

In the ASD group, the lifetime victimisation score was positively correlated with age and the PCL-S scores. Regarding the victimisation score over the previous year, a negative correlation was found with age and a positive correlation was observed with the PCL-S score and the SRS scores. In the control group, a positive correlation was found between the victimisation score over the entire lifetime and the PCL-S score. A negative correlation was found between the victimisation score over the previous year and age while a positive correlation was found with the PCL-S score. All of these correlations were significant in both groups.

**Table 5:** Variables associated with victimisation scores (lifetime and previous year)

	JVQ score/lifetime		JVQ score/previous year	
	ASD (n = 39)	Controls (n = 53)	ASD (n = 39)	Controls (n = 53)
Age	0.307*	0.094	− 0.464**	− 0.276*
PCL-S	0.553***	0.447**	0.341*	0.335
SRS (T-score)	0.042		0.314*	
VCI	0.109		0.049	
FRI	0.136		− 0.147	
IQ	0.074		− 0.001	

Pearson’s correlation. Data are expressed as correlation coefficient

*ASD* autism spectrum disorder, *FRI* fluid reasoning index, *IQ* intelligence quotient, *JVQ* Juvenile Victimization Questionnaire—Screener Sum Version, *PCL-S* post-traumatic stress disorder CheckList—Scale, *SRS* social responsiveness scale, *VCI* verbal comprehension index

* Significance threshold *p* < 0.05. ** Significance threshold *p* < 0.01. *** Significance threshold *p* < 0.001

In the ASD group, we found no significant difference of victimisation depending on gender, the parents’ marital status, the subjects’ type of schooling, the presence of an individual teaching aid or clinical status for ADHD co-morbidity. We found no significant correlation between the victimisation scores and the cognitive profile of IQ sub-scores in the ASD group.

## Discussion

The total score of victimisation and the sub-score of victimisation by peers and siblings were significantly higher in the ASD group than in the TD group. Three quarters of the ASD group have been victimised at school. These results suggest that children and youths with ASD are more severely exposed to victimisation events in general than their typically developing peers, especially peer victimisation at school. Bullying and emotional bullying by peers were significantly more frequent in the ASD group. Nearly 72% of the children and youths with ASD had suffered at least one type of victimisation within the previous year. Nearly 54% had been victimised by their peers or their siblings within the previous year. Twenty-three percent of ASD students were poly-victims. The number of poly-victims did not significantly differ between both groups.

Our results are consistent with the international results found in the literature on ASD and victimisation. Using the JVQ, Little et al. [56] reported a victimisation rate of 94% and a peer victimisation rate of 75% in a population of ASD students [56]. Our results found a victimisation rate of 95% and a peer victimisation rate of 72% in the ASD group with the same questionnaire. Although prevalence estimates of victimisation vary from study to study, a review of 21 articles on prevalence rates of victimisation of school-age children and youths with ASD reported a rate of bullying in this population ranging from 50 to 77%, depending of the type of rating scale (self, teacher or parental reports) and the period of reports (within the last month or over a lifetime) [21]. Figures from a parental survey reported in the UK by the National Autistic Society suggested a rate of victimisation for children with ASD of 40% to 59% [57]. Carter found that 65% of the parents in a sample of children with ASD reported that their children had experienced peer victimisation within the previous year [58]. On a more comparable basis, using smaller samples, Wainscot et al. [59] found that 87% of secondary-age children with ASD or high functioning autism in the UK reported being bullied at least once a week. Cappadocia et al. [14], using parent reporting in a Canadian sample, conducted an online parent-report study of victimisation and mental health among 192 children and adolescents with ASD within the past month. Seventy-seven percent of parents reported that their child had experienced at least one occurrence of victimisation within the past month.

However, there are a number of methodological inconsistencies across studies that make the comparison of results difficult. Reports may vary due to differences in how bullying is defined, the time period under consideration, the methods used (observational vs. questionnaire), and the informants (parent/teacher/self/peer). In addition, we were unable to find any previous results obtained in France. The prevalence of peer victimisation in our French sample is in the high range when compared to other countries, despite variations in scales and informants between studies. Furthermore, our results replicate the previous results of the only previous study using the JVQ [56].

Victimisation was also common among control participants in this study (58.5% victimised within the previous year), in accordance with the prevalence of victimisation found in the general population in the United States (57.7% within the previous year) [36]. According to bullyingstatistics.org, 77% of TD students experience mental, verbal, or physical bullying.

In our study, nearly 68% of control students had been victimised by their peers in their lifetime. This difference in victimisation frequency between the groups was not significant. However, students with ASD were more severely victimised, especially by their peers, than the control students, as the total victimisation score over a lifetime as well as the peer victimisation score within the previous year were both significantly higher in the ASD group.

From a general point of view, it seems that victimisation and bullying should be defined more precisely in the literature. Indeed, depending on the definition of victimisation or the tool assessing victimisation used in the studies, the reported victimisation events include simple teasing and jokes or more serious events such as physical aggressions. The prevalence rate of victimisation may vary depending on which victimisation events are considered.

Co-morbid attention deficit hyperactivity disorder (ADHD) in students with ASD has been associated with peer victimisation in the literature [39, 51]. Although half of our ASD sample had also been diagnosed with ADHD, we did not find a correlation between ADHD comorbidity and higher rates of victimisation, in contrast to previous findings [60–63]. The mean total score of victimisation in our ASD sample might have been too high to highlight a significant correlation. Our results are in accordance with Ashburner et al. [64], who showed that the presence of ADHD was not associated with parental reports on bullying experiences or levels of worry, in contrast to previous findings; however, the reason for this was not clear to the authors.

Our results showed that, among subjects with ASD, younger ones had suffered the most victimisation within the previous year. This result is in accordance with findings in literature [14, 20, 54]. Several studies report rates of bullying that peak during late elementary and middle school years, with a likelihood of being bullied that steadily decreases through middle school and high school [65, 66]. This could suggest a learning process and the development of social adaptation strategies throughout the life of ASD children and youths. Conversely, the oldest individuals had been the most victimised over their entire lifetime, likely due to a cumulative effect of the victimisation events.

The lower the social interaction skills possessed by participants, the more victimised they were according to the significant positive correlation found between victimisation scores and SRS scores. Similar results are found in the literature [52].

Our study highlights the occurrence of PTSD, as PTSD scale scores were significantly higher in the ASD group and more strongly correlated with victimisation rates in this group. The definition of PTSD has recently been updated in the DSM-5 released in 2013. Although PTSD has traditionally been thought to be caused by a single, life-threatening event (or, at least, an event that seemed to be life threatening) [67], in the case of trauma such as bullying, PTSD can also occur due to the accumulation of many small, individually non-life-threatening incidents, referred to as complex PTSD. Complex PTSD is brought on by a series of terrifying events or prolonged, repeated trauma, often in situations where the person has little or no chance of escape. It results in delayed and prolonged symptoms such as anxiety, withdrawal, suicidal behaviour, alcohol and drug abuse, and emotional issues [2].

PTSD prevalence is commonly based on PTSD, as traditionally defined, i.e. caused by a single life-threatening event. The estimated lifetime prevalence of PTSD among adult Americans and Europeans is 7.8%, in Danish Adolescents it is 9% [68, 69]. Three victimised subjects of our ASD sample (8.6%) had a diagnosis of complex PTSD. This result is in accordance with the prevalence of PTSD in the general population but lower than the prevalence of PTSD symptoms found in the literature for ASD students (about 17.4%) [70]. Our prevalence of complex PTSD could have been underestimated due to methodological differences, in particular the use of parental reports versus child reports in previous studies.

PTSD is a complicated issue. Diagnosing this disorder is likely to be difficult in an ASD population. About 40% of children with ASD are diagnosed with at least one comorbid anxiety disorder. Such a diagnosis suggests the presence of excessive worry and fear in the daily life of an ASD child. However, people with PTSD may exhibit excessive fear or hypersensitivities to specific sensory experiences, that is one of the diagnostic criteria for ASD [2]. There can be a phobic reaction to a range of auditory, tactile, visual, and olfactory sensations that will be experienced throughout the day. Anxiety may worsen when such aversive experiences occur [71] and hinder making a diagnosis of PTSD in the ASD population.

Vulnerability to victimisation of ASD children and youths can be explained in part by impairments in social understanding, difficulties with communication and generalisation and higher theory of mind disabilities [2, 72–74]. Regarding individuals with ASD, many researchers have questioned their specific vulnerability and ability to reliably and validly perceive and report bullying and victimisation [20, 21, 75–77]. Theory of mind abilities also predict impaired peer acceptance as difficulties understanding the thoughts, emotions, reactions and behaviours of others impacts the ability of individuals with ASD to monitor feedback from others about how their behaviour is being perceived, which makes them the ideal target for bullying at school [78]. Impairments in understanding feelings and emotions are often suggested as common daily life difficulties in ASD [79]. Rieffe et al. [80] examined the relationship between bullying or victimisation and experiencing basic emotions. Their results suggest that, unlike typically developing children, anger dysregulation plays an important role in victimisation for children with ASD. They propose that this might be related to the emotional reactivity characteristic of many children with ASD. When provoked, students with ASD may display their anger in an overtly visible manner, thus prompting further victimisation.

Most studies suggest that the majority of children and youths who are bullied did not tell an adult at school about it [81]. Prevention and intervention efforts toward the entire student population should be a priority for French school authorities as these interventions will benefit all students, including students with ASD [82]. These specific interventions should be carried out to prevent peer victimisation and its consequences: dropping out, school failure, social self-exclusion, low self-esteem, complex PTSD, and suicide, in severe cases [22, 83]. For example, the Olweus Bullying Prevention Programme (OBPP) [84] is a comprehensive, school-wide programme that was designed to reduce bullying and achieve better peer relations between students in elementary, middle, and junior high school grades. It is the most researched and best-known bullying prevention programme available today. The programme includes school-, classroom-, and individual-level components. The school-level components consist of an assessment of the nature and prevalence of bullying in the school, the formation of a committee to coordinate the prevention programme, and the development of a system ensuring adult supervision of students outside of the classroom. Classroom components include defining and enforcing rules against bullying, discussions and activities to reinforce anti-bullying values and norms and active parental involvement in the programme. Individual components intervene with students with a history of bullying and/or victimisation. Such prevention programmes could help to encourage the disclosure of victimisation events.

Prevention strategies should focus on teaching students with ASD spontaneous communication and age-appropriate social skills to interact successfully with their peers. Peer friendships are important in preventing peer victimisation [29, 31]. Such strategies should aim at building social/emotional competencies, and social networks that secondarily may also reduce the impact of bullying for children and youths with ASD. Conflict-resolution skills are important to develop and sustain lasting peer relationships in adolescence. Social skills training groups and peer programmes may prevent bullying [85]. Peers can be a great resource for both recognising what age-appropriate skills are and supporting children and youths with ASD as they learn those skills. Peer-mediated interventions, such as peer support arrangements and peer networks, have been effective in increasing social interactions between children and youths with ASD and their peers across the school day and within the classroom [86].

One intervention found to be effective at teaching children and youths with ASD friendship skills is the PEERS^®^ programme [87], a parent-assisted social skills group. This programme includes lessons on conversational skills, how to enter and exit a conversation, how to choose appropriate friends, and how to handle teasing and bullying situations. One other intervention showing preliminary evidence of friendship development between students with ASD and their typically developing peers are peer networks [88]. Peer networks are constituted of the different groups of people that we know and who can provide support in the larger social world. In contrast to friendships, which are dyadic, reciprocal relationships with a strong emotional component, peer networks could be defined as peers that we interact with over a period of time. Interventions should also include teaching students how to identify a bullying situation and what to do when it happens [89].

To the best of our knowledge, this is the first controlled study in France assessing the prevalence of victimisation in children and youths with ASD.

All of the measures were based on parental reports because we were not allowed to perform the assessments with the children and youths in this study by French school authorities for ethical reasons. The use of parental reports might have led to measurement bias. Parents of ASD participants benefited from psycho-education from the health professionals of the ASD expert centre and may have been more aware of the victimisation risk, which might have led to an overestimation effect. Also, parenting stress has already been shown to bias the estimate of anxiety in cases of bullying victimisation among adolescents with ASD [90]. There is considerable methodological variability among studies assessing victimisation in ASD students. Further studies should be conducted in order to develop shared rating methods and informant selection criteria.

Other limitations include possible sampling biases. ASD children and youths were recruited from a specialised diagnostic centre in France, which may not be representative of the ASD general population. French school authorities allowed us to intervene in only one private regular schooling institution, which may not be representative of the French students’ population.

The fact that no parent has reported the presence of ADHD in the control group should be discussed, but the reason is unclear. This may be due to the design of the study (no clinical assessment in the control group) and/or the fact that parents may have been reluctant to report such a disorder at school, even though the data collection was completely anonymous.

Although we chose to explore victimisation in its various aspects, bullying remained the main focus of this study. Conversely, some forms of victimisation, like sexual victimisation—which is more common in girls [91, 92]—might have been underestimated due to a predominantly male sample.

## Conclusion

Children and youths with ASD are more vulnerable to peer victimisation, especially bullying, at school. The younger they are and the lower their level of social skills, the more severe the victimisation is. Moreover, our results highlight the occurrence of complex PTSD in these children and youths who are victims. These preliminary results call for further multi-centred studies in larger samples and research into more specific tools for assessing victimisation in children and youths with ASD.

There is also room for improvement in the assessment and prevention of anxiety in children and youths with ASD, especially complex PTSD.

As the trend of mainstreaming schoolchildren with disabilities increases, greater comprehension of bullying issues in the ASD population is urgently needed in order to minimize these events and their consequences. Findings might lead to better teacher training and the development of effective peer victimization prevention strategies, which need to be included in the French educational curriculum.

Some interesting training sessions have been tested in France but no global and validated programmes are currently available. These common training pilot sessions are based on the pivotal response training (PRT) that has been proposed by some therapists in France [93], which was inspired by the social interaction skills training manual [94]. PRT is a naturalistic form of applied behaviour analysis [95] used as an intervention for children with autism, which was pioneered by Robert and Lynn Koegel [96]. PRT advocates that behaviour hinges on “pivotal” behavioural skills—motivation and the ability to respond to multiple cues—and that the development of these skills will result in collateral behavioural improvements. In 2005, Richard Simpson of the University of Kansas identified pivotal response treatment as one of the four scientifically-based treatments for ASD [97]. These pilot training sessions in French schools seem to highlight some interesting components: age of the peers involved (who should be older than the child with ASD), type of activity (outdoor and motors activities preferably), and motivation and modelling components that could be considered for future intervention programmes.

However, larger and more structured programmes, such as the PEERS^®^ programme, need to be translated into French and validated in our community. This programme was originally developed by Dr Elizabeth Laugeson at UCLA in the United States [87]. It offers basic guidelines for motivated French students to develop social skill interventions to help ASD children and youths to make and maintain friendships. Nevertheless, the development of the role of school and social assistants in French schools is one of the main French projects aimed at promoting inclusive education and kindness at school [98]. French school action plans should also include the promotion of a culture and climate that is welcoming to diversity and where teachers and assistants are trained to manage relationships and social skills.

## Abbreviations

ADI-R: autism diagnostic interview-revised
ADHD: attention deficit hyperactivity disorder
ADOS-G: autism diagnostic observation schedule-generic
ASD: autism spectrum disorder
DSM: diagnostic and statistical manual
ID: intellectual disability
IQ: intelligence quotient
JVQ: Juvenile Victimization Questionnaire—Screener Sum Version
PCL-S: Post-traumatic stress disorder CheckList-Scale
PRT: pivotal response training
PTSD: post-traumatic stress disorder
SD: standard deviation
SRS: social responsiveness scale
TD: typically developing
